# JUN activation modulates chromatin accessibility to drive TNFα‐induced mesenchymal transition in glioblastoma

**DOI:** 10.1111/jcmm.17490

**Published:** 2022-07-18

**Authors:** Xuejiao Lv, Qian Li, Hang Liu, Meihan Gong, Yingying Zhao, Jinyang Hu, Fan Wu, Xudong Wu

**Affiliations:** ^1^ State Key Laboratory of Experimental Hematology, The Province and Ministry Co‐sponsored Collaborative Innovation Center for Medical Epigenetics, Key Laboratory of Immune Microenvironment and Disease (Ministry of Education), Department of Cell Biology Tianjin Medical University Tianjin China; ^2^ Department of Neurosurgery Tongji Hospital, Tongji Medical College, Huazhong University of Science and Technology Wuhan China; ^3^ Department of Molecular Neuropathology Beijing Neurosurgical Institute, Capital Medical University Beijing China; ^4^ Department of Neurosurgery Tianjin Medical University General Hospital Tianjin China

**Keywords:** AP‐1, chromatin accessibility, glioblastoma, JUN, mesenchymal transition, TNFα

## Abstract

Chromatin dynamics as well as genetic evolution underlies the adaptability of tumour cells to environmental cues. Three subtypes of tumour cells have been identified in glioblastoma, one of the commonest malignant brain tumours in adults. During tumour progression or under therapeutic pressure, the non‐mesenchymal subtypes may progress to the mesenchymal subtype, leading to unfavourable prognosis. However, the molecular mechanisms for this transition remain poorly understood. Here taking a TNFα‐induced cellular model, we profile the chromatin accessibility dynamics during mesenchymal transition. Moreover, we identify the JUN family as one of the key driving transcription factors for the gained chromatin accessibility. Accordingly, inhibition of JUN phosphorylation and therefore its transcription activity successfully impedes TNFα‐induced chromatin remodelling and mesenchymal transition. In line with these findings based on experimental models, JUN activity is positively correlated with mesenchymal features in clinical glioblastoma specimens. Together, this study unveils a deregulated transcription regulatory network in glioblastoma progression and hopefully provides a rationale for anti‐glioblastoma therapy.

## 1 INTRODUCTION

Glioblastoma is the most common primary malignant brain tumour in adults and is fatal on account of its invasive properties. The current standard care of glioblastoma patients is maximal surgical resection, adjuvant chemo‐radiotherapy and neoadjuvant immunotherapy. So far, no efficacious treatment has been demonstrated to significantly increase median lifespan (12–18 months).^1^ One of the main causes of poor prognosis lies in high cellular plasticity and immense intratumoral heterogeneity.^2^, ^3^, ^4^, ^5^, ^6^ Strategies to restrict cellular plasticity have been shown to suppress glioblastoma progression.^7^, ^8^

To dissect this lethal disease at the molecular level, great efforts have been put into the genomic and transcriptomic profiling of glioblastomas in the past two decades.^9^, ^10^, ^11^ As the first cancer type of The Cancer Genome Atlas (TCGA) pilot projects, glioblastomas have been categorized into three subtypes, namely proneural (PN), classical (CL), and mesenchymal (MES), according to integrated analyses of multi‐omics data.^12^, ^13^ These molecular profiles have played important guiding roles in prognosis and therapeutics. Generally speaking, the MES subtype is associated with worse prognosis than the other two subtypes, considering of its invasiveness, escape from immunosurveillance or therapeutic resistance.^14^, ^15^, ^16^, ^17^

Nevertheless, emerging evidence from single‐cell RNA sequencing analyses has demonstrated that multiple subtypes may co‐exist even in a single glioblastoma sample.^2^, ^5^, ^18^ These subclones may evolve from different genetic background or epigenetic changes. Interestingly, isolation and in vitro culture of patient‐derived glioblastoma stem cells (GSCs) in optimal conditions generally recapitulate their unique transcriptomic signature.^19^, ^20^ For examples, CD44 has been frequently used as a MES marker, whereas OLIG2 expression is typical of PN‐subtype.^19^, ^20^ However, their biological characteristics are not perfectly maintained in vitro, especially for the MES subtype. The loss of MES features may be due to the absence of signals from tumour microenvironment. Indeed, the addition of tumour necrosis factor α (TNFα), a well‐known inflammatory cytokine, induces MES features in GSCs.^19^ Consistently, the genes of TNFα receptor superfamily and the NF‐kB pathway are enriched in the MES subtype of tumours. Moreover, PN or CL subtype may transform from non‐MES to MES subtype (mesenchymal transition, MT) in unfavourable inflammatory conditions due to hypoxia, necrosis or in adaption to chemoradiotherapies.^14^, ^16^, ^19^, ^21^, ^22^ Hence, it is critical to understand the molecular basis of MT.

Epigenetic changes are the main underlying causes of cell fate transitions, for either normal development or malignant transformation. Here, by comparing chromatin accessibility in a TNFα‐driven MT model, we find that the chromatin regions with gained accessibility in the induced MES subtype are enriched with JUN transcription factor family. Moreover, we show that inhibition of JUN phosphorylation prevents the gain of MES‐specific accessibility and reverts TNFα‐induced MT process. Therefore, JUN transcription regulatory network is necessary to remodel chromatin accessibility and thereby promotes glioblastoma progression, which will hopefully provide a novel targeting therapeutic strategy.

## 2 MATERIALS AND METHODS

### 2.1 Cell culture and compounds

The glioblastoma cell line TPC 2–4 was obtained from Beijing Tiantan Hospital and were cultured in DMEM/Ham's F12 (12400024, Gibco) supplemented with 2% B27 Supplement (17504044, Gibco), 1% P/S, 1% L‐Glutamine 200 mM (100×, 25030081, Gibco), 20 ng/ml human fibroblast growth factor‐basic (bFGF, 10014‐HNAE, Sino Biological) and 20 ng/ml epidermal growth factor‐basic (EGF, 10605‐HNAE, Sino Biological). Cells were maintained at 37°C in a humid incubator with 5% CO_2_. Cells have been tested for mycoplasma contamination by PCR and were verified to be mycoplasma free. TNFα was purchased from Sino Biological (10602‐HNAE, while JNK‐IN‐8 from MCE (HY‐13319), and both compounds were dissolved in dimethyl sulfoxide (DMSO, D8418, Sigma).

### 2.2 Immunohistochemical staining and analysis

The paraffin‐embedded glioblastoma tissue microarray (TMA) was used for IHC staining,^23^ and 40 cases of WHO grade IV glioblastomas were further analysed. The slide was performed with de‐paraffinized, rehydrated, and antigen retrieval, then blocked for at least 1 h at 37°C with 1% BSA, and then incubated overnight at 4°C with primary antibodies (anti‐c‐Jun (60A8) Rabbit mAb (1:100, CST, 9165S), anti‐c‐Jun (phospho S63) antibody [Y172] (1:100, Abcam, ab32385), anti‐CD44 Polyclonal antibody (1:100, Proteintech, 15,675‐1‐AP) and anti‐OLIG2 antibody [EPR2673] (1:100, Abcam, ab109186)). After careful washing with 1 × PBS, the slides were incubated with horseradish peroxidase (HRP)‐conjugated antibodies, DAB was used for chromogenic reaction, and the nuclei were counterstained with haematoxylin. The TMA images were taken by Vectra Polaris Automated Quantitative Pathology Imaging System. In addition, we quantitatively scored the tissue sections according to the percentage of positive cells and staining intensity. Tissues too small and/or crushed on the TMA were eliminated from analysis.

### 2.3 Transwell assay

Twenty‐four‐well Transwell chambers with 8‐mm pore size (Corning Costar) were used to perform migration assay. The upper chamber was inoculated with 5 × 10^4^ TPC2‐4 cell suspension (control, TNFα‐induced cells treated with or without JNK‐IN‐8) re‐suspended with DMEM/F12. The lower chamber was filled with 5% foetal bovine serum contained‐DMEM/F12 medium. Cells in the upper chamber were carefully removed 24 h later. The migrated and invaded cells in lower chamber medium were imaged and calculated.

### 2.4 RNA extraction and RT‐qPCR analysis

As previously described,^24^ the extracted RNAs were subjected to reverse transcription with DNase I (10104159,001, Sigma), RevertAid Reverse Transcriptase (EP0442, Thermo Scientific) and Ribolock Rnase inhibitor (EO0382 EO0382). RT‐qPCR reactions were performed with 2× ChamQ Universal SYBR qPCR Master Mix (Q711‐03, Vazyme) on LightCycler480 II (Roche). *rPO* was used as a reference gene for all qRT‐PCR experiments and analyses. The ΔΔCt method was used for quantification analysis. Gene‐specific primers were as follows:

hTNFAIP3 forward: 5′‐TCAACTGGTGTCGAGAAGTCC‐3′,
hTNFAIP3 reverse: 5′‐ CAAGTCTGTGTCCTGAACGC‐3′;
hNFKBIA forward: 5′‐ GTCCTTGGGTGCTGATGT‐3′,
hNFKBIA reverse: 5′‐ GAGAATAGCCCTGGTAGGT‐3′;
CD44 forward: 5′‐ AGGAGACCAAGACACATTCCAC‐3′,
CD44 reverse: 5′‐ CACCTTCTTCGACTGTTGACTG‐3′;
CDH1 forward: 5′‐GAAATCACATCCTACACTGCCC‐3′,
CDH1 reverse: 5′‐GTAGCAACTGGAGAACCATTGTC‐3′.

### 2.5 RNA sequencing (RNA‐seq) and data analysis

Total RNA was extracted with Trizol Reagent (15596018, life) according to the instructions. RNA‐seq libraries were sequenced on the Illumina PE150 platform by Novogene. The data analysis was carried out as described.^25^ Reads were aligned to the hg19 genome using TopHat v2.0.6 with the library type option set to first strand. FragmentsPer Kilobase per Million (FPKM) values for known genes were calculated using Cufflinks v2.0.2 provided with the GTF file via the ‐G (known genes only) option. FPKM values were quantile normalized.

### 2.6 ATAC sequencing and data analysis

For ATAC‐seq, 5 × 10^4^ TPC2‐4 cells were precipitated to prepare samples according to previous description.^26^ Briefly, cell pellets were re‐suspended with 50 μl cold lysis buffer (10 mM Tris–HCl pH 7.5, 10 mM NaCl, 3 mM MgCl_2_, 0.1% [v/v] igepal CA‐630) and incubated on ice for 10 min. After centrifuge at 500 *g* for 10 min at 4°C, the nuclei were re‐suspended with 100 μl wash buffer (10 mM Tris–HCl pH 7.5, 10 mM NaCl, 3 mM MgCl_2_). Followed by washing twice, the transposable reaction was performed. Subsequently, DNA library was generated accordingly with TruePrep DNA Library Prep Kit V2 (TD501‐01, Vazyme). The successfully prepared libraries were sequenced on the Illumina PE150 platform (Novogene).

### 2.7 Gene‐set enrichment analysis

Gene‐set enrichment analysis (GSEA) was performed with the public application from the Broad Institute. FKPM values for all human genes generated from RNA‐seq data were used for expression datasets. KEGG pathway gene sets were used for analysis. False discovery rate (FDR) was calculated by repeating sample permutations 1000 times. To be able to subtype GSC lines, a single sample gene set enrichment analysis (ssGSEA) was performed using 50‐gene signatures for each subtype as defined previously.^13^

### 2.8 Statistical analysis

All grouped data are presented as mean ± SD. Unpaired Student's *t*‐tests are presented as mean ± SD during comparison between unpaired two groups, and one‐way anova was applied for multi‐group data comparison. Bivariate correlation analysis (Pearson's *r* test) was used to examine the correlation of two variables in human specimens.

## 3 RESULTS

### 3.1 TNFα induces MT of glioblastoma cells

**FIGURE 1:**
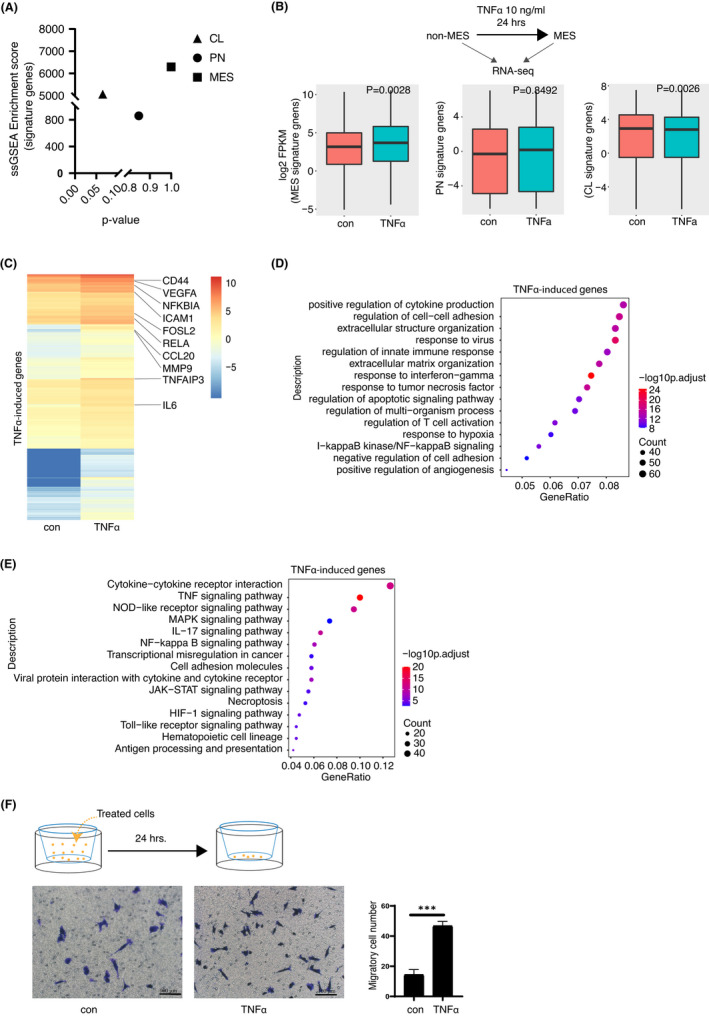
TNFα induces MES transition of non‐MES glioblastoma cells. (A) The ssGSEA enrichment score of TPC2‐4 and the statistics. (B) Upper panel shows the schematic of in vitro MT models. Boxplots to compare the differential expression levels of MES, CL or PN subtype 50‐gene signatures in TPC2‐4 cells treated with or without TNFα. (C) Heatmap shows the 830 transcripts of TNFα‐induced genes (fold change [TNFα/control] ≥1.5). Representative genes are marked. (D–E) GO (D) and KEGG pathway (E) enrichment of TNFα‐induced genes. Tops of enriched pathway are shown. (F) Transwell assay was performed using TPC2‐4 suspension treated with TNFα (10 ng/ml), respectively. Migrated cells were counted and compared. Error bars ± SD, *n* = 3. Images are representatives of three independent experiments. Scale bar 100 μm. Two‐tailed unpaired Student's *t*‐test was performed for F, ***p < 0.001.

To establish an in vitro cellular model for MT, we took advantage of a patient‐derived GSC TPC2‐4, which was validated closely resemble to CL subtype according to its transcriptome profiles (the single sample gene set enrichment analysis (ssGSEA) score of CL subtype was 5,069.88, *p*‐value 0.0649; ssGSEA‐value of PN was 861.15, *p*‐value 0.8481; ssGSEA‐value of MES was 6293.51, *p*‐value 1) (Figure 1A). By treating this cell line with TNFα for different concentrations (0, 5, 10, 20 and 50 ng/ml) and for different duration (12, 24, 36 and 48 h), we measured the expression levels of several marker genes through RT‐qPCR analysis. As shown in Figure S1A–C, TNFα (10 ng/ml) for 24 h is sufficient to induce significantly upregulated expression of *TNF Alpha Induced Protein 3* (*TNFAIP3*), NF‐κB pathway‐associated genes *Nuclear Factor Kappa B Subunit 1* (*NFKB1A*) and the MES marker gene *CD44* and significantly downregulated expression of epithelia marker gene *CDH1* (*E‐CAD*).

When we compared the transcriptomic changes at this condition through RNA‐seq analysis, we found that the expression levels of defined mesenchymal genes were significantly induced by TNFα treatment, while a significant decrease of CL signature gene expression was observed (Figure 1B). In addition, as shown by heatmap in Figure 1C, the expression levels of 830 genes were significantly increased in GSCs after treatment with TNFα (fold change of FPKM >1.5). Gene Ontology (GO) analysis of these upregulated genes showed that TNFα‐induced genes are significantly enriched in the regulation of cell–cell adhesion, extracellular matrix organization and response to tumour necrosis factor (Figure 1D), indicative of increased aggressiveness. In addition, the Kyoto Encyclopedia of Genes and Genomes (KEGG) analysis showed that the TNFα signalling pathway, NOD‐like receptor signalling pathway and NF‐kappa B signalling pathway are significantly activated upon TNFα treatment (Figure 1E). To confirm the phenotypic changes, we performed in vitro transwell assay. Briefly, either control of the TNFα‐treated TPC2‐4 cells were incubated in a growth factor‐free medium in the upper chamber and allowed to migrate to the lower chamber filled with regular culture medium. As shown in Figure 1F, TNFα‐treated cells showed significantly stronger migration abilities than the control cells. Overall, these data indicate that TNFα treatment of glioblastoma cells nicely recapitulates MT process.

### 3.2 TNFα‐induced chromatin remodelling

**FIGURE 2:**
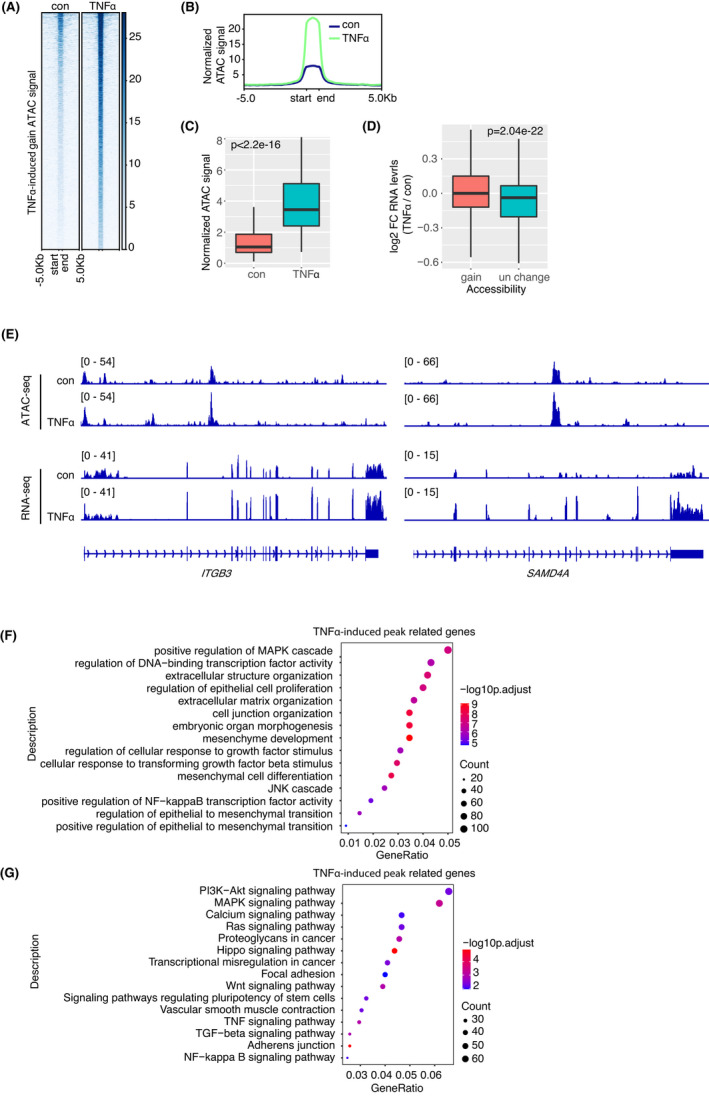
TNFα induced chromatin remodelling during MES transition. (A–C) Heatmap (A), profiles (B) and densities (C) of ATAC signals at gain regions (from start to end of associated genes) in TPC2‐4 cells treated with TNFα (10 ng/ml) versus control. (D) Corresponding mRNA expression levels of associated genes with ATAC signal gain in TPC2‐4 cells treated with TNFα versus control. The gene without significant changes of ATAC signals was served as negative controls. (E) Genomic snapshots of ATAC‐seq and RNA‐seq analyses of *ITGB3* and *SAMD4A* gene in TPC2‐4 cells treated with TNFα versus control. The y‐axis represents the normalized number of reads. (F–G) GO (F) and KEGG pathway (G) enrichment of associated genes with ATAC signal gain. Tops of enriched are shown.

Epigenetic dysregulation is a defining feature of tumorigenesis and tumour progression.^27^, ^28^, ^29^ To better understand the genome‐wide alterations of chromatin accessibility in GSCs after treatment with TNFα, we performed comparative analysis of chromatin accessibility using the assay for transposase accessible chromatin with sequencing (ATAC‐seq). As shown by heatmap and profiles in Figure 2A and B, chromatin accessibility at thousands of regions was strikingly increased in GSCs after treatment with TNFα (3673 peaks with fold change >2). To further quantify the changes, we compared the average ATAC signal densities at these chromatin regions in the two groups. As shown by the boxplot, the increase of chromatin accessibility in TNFα‐induced group is indeed significant (Figure 2C). As expected, the expression levels of associated genes with gained accessibility are significantly upregulated in GSCs treated with TNFα, compared with the ones without significant changes of chromatin accessibility (Figure 2D). For instance, the chromatin accessibility of the upregulated gene *ITGB3* and *SAMD4A* is significantly increased in GSCs after being treated with TNFα (Figure 2E). In addition, this chromatin remodelling does not occur randomly. GO analysis showed that the associated genes with these regions of gained accessibility are primarily enriched for extracellular structure organization, mesenchyme development and regulation of epithelial to mesenchymal transition (Figure 2F). Meanwhile, KEGG analysis demonstrated that these genes are significantly associated with signalling pathways that play vital roles in tumour progression and metastasis, for instance, PI3K‐AKT, MAPK and Ras signalling pathways (Figure 2G). Thus, TNFα‐induced chromatin remodelling is consistent with the functional MT process.

### 3.3 JUN activation is necessary for TNFα‐induced accessibility gain

**FIGURE 3:**
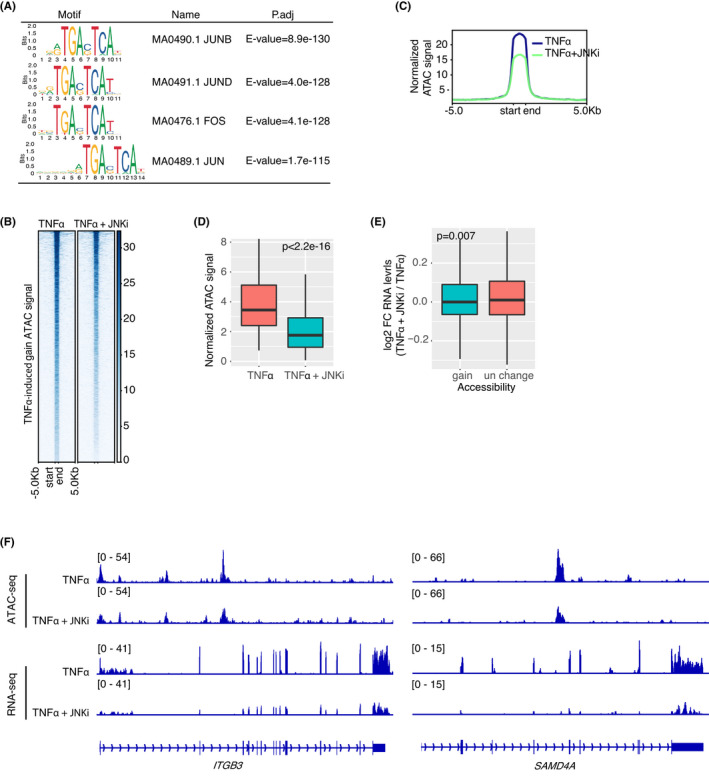
JUN activation is necessary for TNFα‐induced accessibility gain. (A) Motif enrichment analyses of chromatin regions with TNFα induced gain of accessibility. Tops of enriched are shown. (B–D) Heatmap (B), profiles (C) and densities (D) of ATAC signals at TNFα‐induced chromatin regions with gained accessibility with or without JNK‐IN‐8 treatment (1 μM). (E) Corresponding mRNA expression levels of JUN‐dependent TNFα‐induced genes. (F) Genomic snapshots of ATAC‐seq and RNA‐seq analyses of *ITGB3* and *SAMD4A* gene in TNFα‐induced cells with or without JNKi treatment. The y‐axis represents the normalized number of reads.

To identify the potential driving factors for MT‐associated chromatin remodelling, we performed de novo motif analysis for the potential binding transcription factors at the TNFα‐induced chromatin regions with gained accessibility. Unexpectedly, the motifs with the most significant enrichment are binding sequences for JUN transcription factor family, rather than NF‐κB (Figure 3A). JUN family proteins are proto‐oncoproteins and critical regulators of cellular proliferation, apoptosis and tumorigenesis.^30^, ^31^ The N‐terminal phosphorylation by Jun N‐terminal kinases (JNK) stimulates its transcription activity.^32^, ^33^ Thus, the downstream signalling pathways of TNFα induction like MAPK/JNK pathway may play dominant roles in chromatin remodelling through activation of JUN family proteins.

To confirm this hypothesis, we simultaneously treated TNFα‐induced TPC2‐4 cells with JNK‐IN‐8 (JNKi), a kinase inhibitor specific for JNK that phosphorylates c‐JUN.^34^, ^35^, ^36^ As demonstrated by ATAC‐seq analyses, a large subset of chromatin regions with TNFα‐induced gain accessibility became less open when the TNFα‐induced cells were treated with JNKi for 24 h (Figure 3B–D). Meanwhile, RNA‐seq analyses showed that the expression levels of TNFα‐induced genes were specifically downregulated upon treatment of JNKi, whereas the expression levels of genes irresponsive to TNFα remained constant (Figure 3E). For examples, the chromatin accessibility and expression levels of the TNFα‐induced gene *ITGB3* and *SAMD4A* are strikingly decreased in GSCs after JNKi treatment (Figure 3F). Taken together, these data demonstrate that JUN activation is necessary for TNFα‐induced chromatin remodelling and upregulated expression of associated genes.

### 3.4 Inhibition of JUN activation impedes TNFα‐induced MT

**FIGURE 4:**
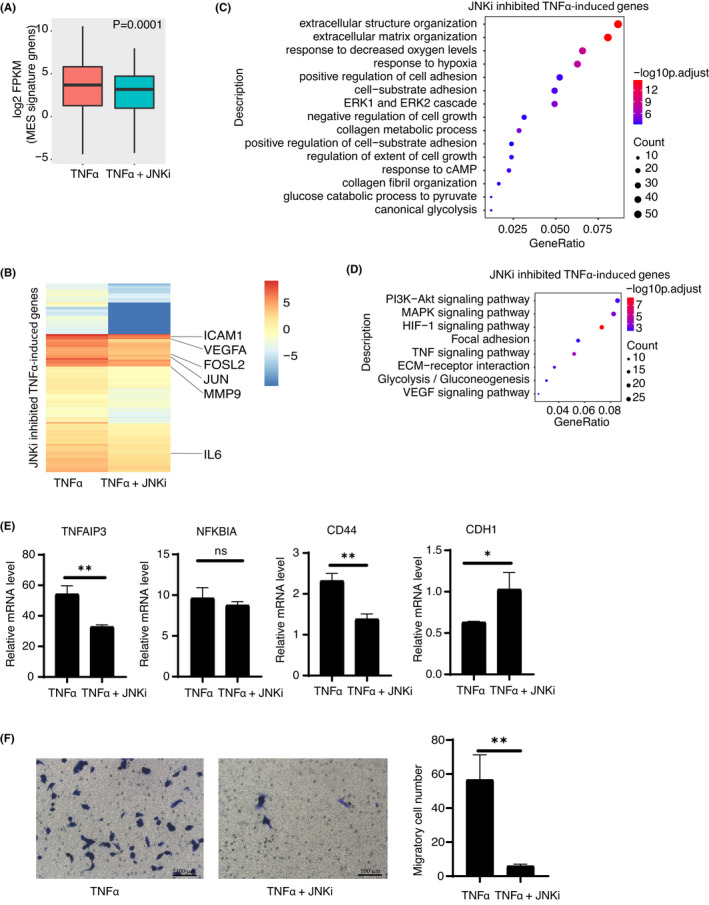
Inhibition of JUN activation impedes TNFα induced MT. (A) Boxplot to compare the differential expression levels of mesenchymal subtype 50‐gene signatures in TNFα‐induced cells treated with or without JNK‐IN‐8. (B) Heatmap shows the 268 transcripts of JNKi inhibited TNFα‐induced genes in TNFα‐induced cells with or without JNKi treatment. Representative genes are marked. (C–D) GO (C) and KEGG pathway (D) enrichment of differentially expressed genes in TNFα‐induced cells treated with or without JNK‐IN‐8. Tops of enriched pathway are shown. (E) RT‐qPCR analysis of the mRNA expression levels of *TNFAIP3*, *NFKB1A*, *CD44* and *CDH1* in TNFα‐induced cells treated with or without JNK‐IN‐8. Error bars ± SD, *n* = 3. (F) Transwell assay was performed using TNFα‐induced cells treated with or without JNK‐IN‐8. Migrated cell was counted and compared. Images are representatives of three independent experiments. Scale bar 100 μm. Two‐tailed unpaired Student's *t*‐test was performed for E and F and erro bars mean ± SD, *p < 0.05, **p < 0.01, n.s. non‐significant, *n* = 3.

We next asked whether inhibition of JUN activity reverses TNFα‐induced MT. Through RNA‐seq analysis, we found that the expression levels of MES signature genes were significantly decreased after JNKi treatment, whereas the expression of PN or CL signature genes was not significantly altered (Figure 4A, Figure S2). Among 830 TNFα‐induced genes, the expression levels of 268 genes are significantly downregulated after JNKi treatment (fold change of FPKM <2/3) (Figure 4B). GO analysis showed that these genes suppressed by JNKi were associated with extracellular matrix or structure organization, and response to decreased oxygen levels (Figure 4C), which suggests that TNFα‐driven malignant transformation could be attenuated by inhibition of JUN activity. In addition, the KEGG analysis showed that the activation of PI3K‐AKT, HIF‐1 and TNF signalling pathways were significantly affected upon inhibition of JUN activity (Figure 4D). Through independent RT‐qPCR analyses, we found that the TNFα‐induced deregulation of representative gene expression was significantly restored by JNKi (Figure 4E). Moreover, JNKi treatment of U87 (a human MES‐subtype cell line) also proved that JNKi inhibition affected the maintenance of MES expression features (Figure S3). In line with the gene expression changes, in vitro transwell assay demonstrated that the invasive capability of TNFα‐induced cells was significantly attenuated by JNKi treatment (Figure 4F). In addition, transwell assay of U87 and 3399 cells (a home‐made mouse MES‐subtype of glioblastoma cell line induced with *kRas* ^mu^/*P53* ^−/−^ adenovirus) showed that JNKi significantly decreased the migratory rate of both cells (Figure S4). Therefore, JUN activation is indispensable for TNFα‐induced MT and even for the maintenance of MES‐like biological features. These data also prompt us to further examine the therapeutic efficacy of JNKi in glioblastoma xenograft models in the future, potentially in combination with conventional chemoradiotherapies.

### 3.5 JUN activation is correlated with MES features in human glioblastoma specimens

**FIGURE 5:**
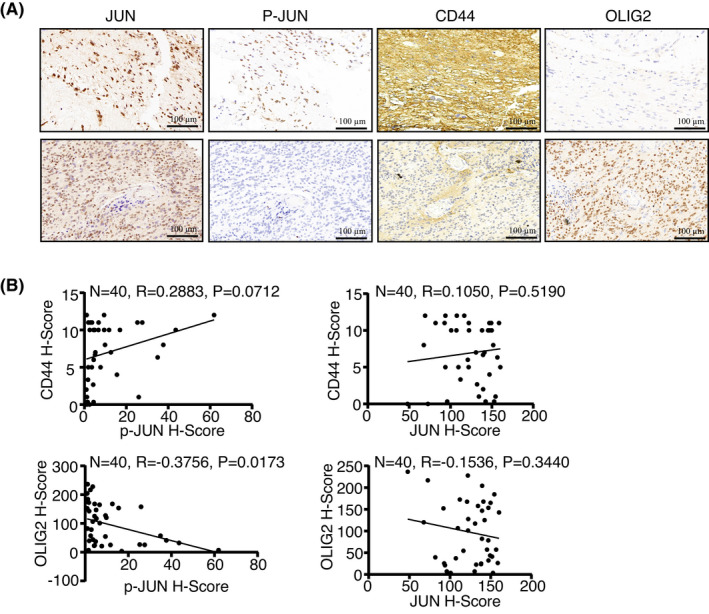
JUN activation is correlated with MES features in human glioblastoma specimens. (A) Tumour sections from 40 glioblastoma specimens (WHO grade IV) were IHC‐stained with anti‐c‐JUN, anti‐c‐JUN (phospho S63), anti‐CD44 and anti‐OLIG2 antibodies. Representative images are shown. Scale bar, 100 μm. (B) Stainings of each target protein were scored, and the significance of the correlation was determined by Pearson's product–moment correlation test (two‐sided).

To confirm that the reprogrammed TF network during MT is indeed clinically significant, we performed immunohistochemical (IHC) staining in a glioblastoma tumour microarray with 40 clinical WHO grade IV glioblastoma specimen serial paraffin‐embedded sections.^23^ The protein expression levels of CD44, OLIG2, c‐JUN and the phosphorylation levels of c‐JUN (pJUN) were analysed. Interestingly, the samples with OLIG2^low^ and CD44^high^ (MES signature) were significantly coincided with p‐JUN positivity (the correlation coefficient [*R*] of CD44 and p‐JUN was 0.2883, *p*‐value 0.0712; *R*‐value of OLIG2 and p‐JUN ‐0.3756, *p*‐value 0.0173), while the significant correlation with expression levels of total c‐JUN was not detected (Figure 5A and B). Overall, these data suggest that the elevated activity of JUN is positively correlated with MES‐subtype of pathological features in human glioblastoma.

## 4 DISCUSSION

Cell plasticity such as epithelial‐mesenchymal transition (EMT) in solid cancers has been a well‐recognized mechanism for tumour progression and often occurs upon treatment and recurrence, which predicts poor prognosis and treatment resistance. Emerging evidences link chromatin changes to this cell reprogramming.^37^, ^38^ In this study, we focus on MT in glioblastoma and dissect the reconfigured transcription factor regulatory network. Excitingly, targeting the driving factors responsible for this chromatin remodelling efficiently reduces invasiveness of glioblastoma.

Actually, numerous previous studies have characterized transcription factors that may drive MT in glioblastoma. For instance, STAT3 and C/EBPβ as master regulators of MT were associated with increased tumour infiltration and glioblastoma recurrence.^39^ SOX10 repression remodels the glioblastoma enhancer landscape and promote MT process.^40^ Here, our study adds another well‐known proto‐oncoprotein JUN as one of the drivers in the context of TNFα. Jun is a component of the ubiquitously expressed heterodimeric transcription factor activating protein 1 (AP‐1) that is rapidly activated in response to numerous extracellular signals.^30^, ^31^ Consistent with our findings, the expression of FOSL1, another AP‐1 comprising protein, has been found to be associated with mesenchymal features and promote aggressiveness in glioblastoma.^41^ Interestingly, a recent study carefully profiled the transcriptomic changes during gliomagenesis at the resolution of single cells and found that AP‐1 is one of the key hubs that triggers a burst of oncogenic alterations for tumour progression. In line with this, transient early‐stage AP‐1 inhibition is sufficient to inhibit gliomagenesis in vivo and provide survival benefits.^42^ Therefore, ours and other studies have mapped a complexed regulatory network linked by cascades of transcription factors for MT in glioblastoma progression. In addition, it warrants further studies to clarify how these networks may interplay in complexed tumour microenvironment.

In addition to transcription factors, chromatin regulators actively participate in these cell fate transitions. However, their pathological functions are generally context‐specific. For instance, though Polycomb group proteins have been well known to be deregulated in malignancies, it seems that their roles differ in difference cancers and even at different stages of the same cancer type.^43^, ^44^ Their core members such as EZH2 and BMI1 seem to divide their work in the PN and MES subtype of glioblastoma. Given that glioblastoma is highly heterogenous with distinct subtypes, combined inhibition of BMI1 and EZH2 provide much stronger anti‐tumour efficacy than targeting each one alone.^20^ Though EZH2 inhibition has been approved by FDA for the treatment of a few cancer types,^44^ prolonged EZH2 depletion in glioblastoma even causes cell fate switch and results in tumour progression.^45^ So far it remains unclear of the long‐term effects of BMI1 inhibition alone in anti‐glioblastoma treatment.^46^ Accordingly, further studies are required to carefully characterize potential lineage switches during tumour progression. Comprehensive understanding the roles of transcription factors, epigenetic regulators at specific extracellular contexts, will be helpful to improve therapeutic precision and efficacy.

In conclusion, this study demonstrates that activated JUN is necessary and sufficient to remodel chromatin accessibility and trigger the activation of mesenchymal subtype‐specific transcription regulatory network, and thereby promotes glioblastoma progression. Considering of the overall activation of AP‐1 in solid cancers, it will be interesting to find out whether it has broader significance in cellular plasticity beyond glioblastoma.

## AUTHOR CONTRIBUTIONS

**Xuejiao Lv:** Data curation (lead); formal analysis (lead); methodology (lead); writing – original draft (equal). **Qian Li:** Formal analysis (equal); visualization (equal). **Hang Liu:** Methodology (equal). **Meihan Gong:** Formal analysis (equal). **Yingying Zhao:** Formal analysis (equal). **Jinyang Hu:** Resources (supporting). **Fan Wu:** Resources (supporting). **Xudong Wu:** Conceptualization (lead); funding acquisition (lead); writing – original draft (equal); writing – review and editing (lead).

## CONFLICT OF INTEREST

The authors declare no conflicts of interest.

## Supporting information


Appendix S1

## Data Availability

The RNA‐seq and ATAC‐seq data that support the findings of this study have been deposited in the Gene Expression Omnibus (GEO) under accession GSE194222.
