# Supplementary material for: JUN activation modulates chromatin accessibility to drive TNFα‐induced mesenchymal transition in glioblastoma
Source: J Cell Mol Med. 2022 Jul 18;26(16):4602–12. doi: 10.1111/jcmm.17490 (PMC9357637; doi:10.1111/jcmm.17490)
Supplement: Supplementary file 1 — Appendix S1 [file JCMM-26-4602-s001.pdf]

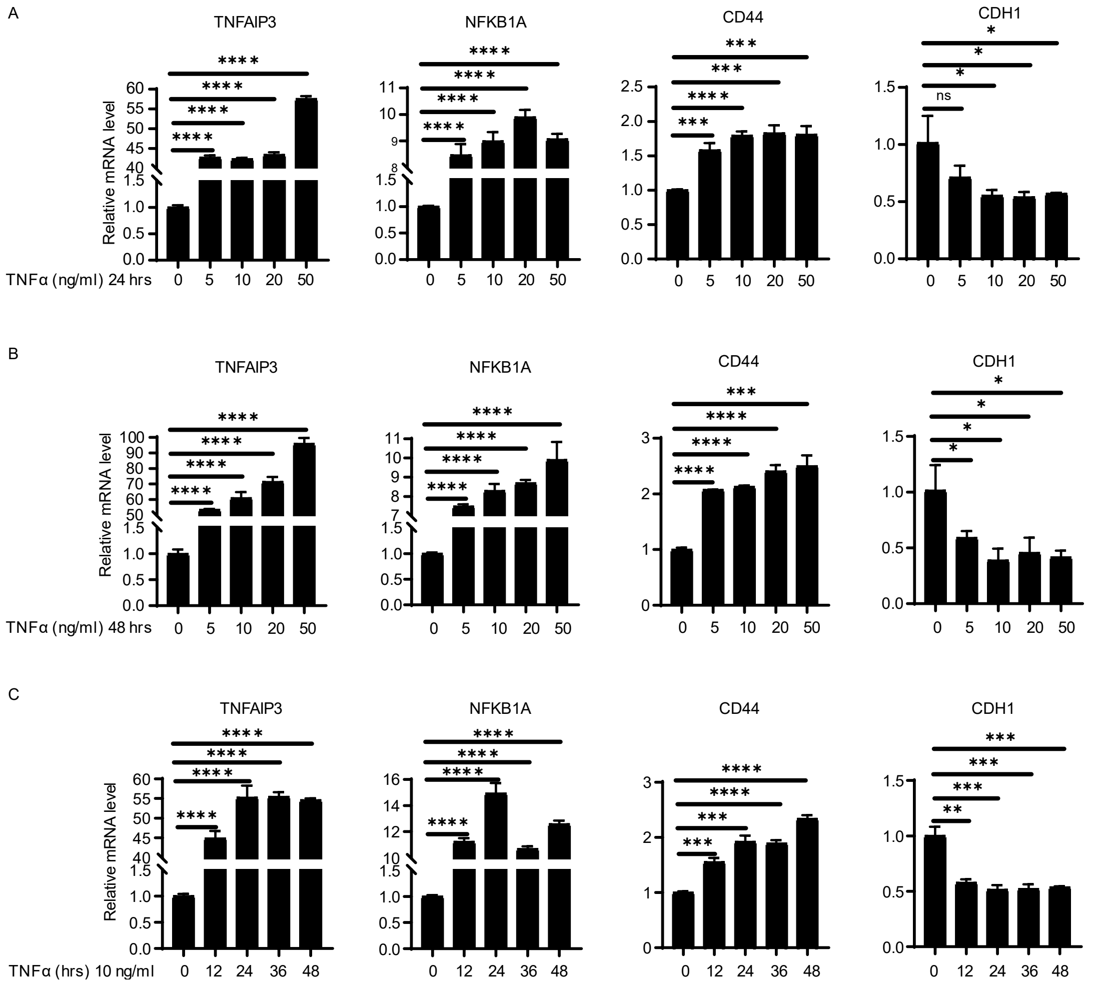

Supplementary Figure 1. Optimization of concentration and time gradient for TNFα treatment of TPC2-4 cells. (A & B) Concentration optimization of TNFα treatment. (C) Time optimization of TNFα treatment (10 ng/ml). Two-tailed unpaired student's *t*-test was performed, Error bars ± SD, * *P*< 0.05,** *P*< 0.01, *** *P*< 0.001, **** *P*< 0.0001. n.s. non-significant, n=3.

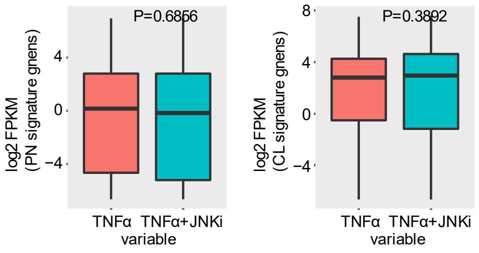

Supplementary Figure 2. Inhibition of JUN activation impedes TNFα induced MT. Boxplots to compare the differential expression levels of CL or PN subtype 50-gene signatures in TNFα-induced cells treated with or without JNK-IN-8.

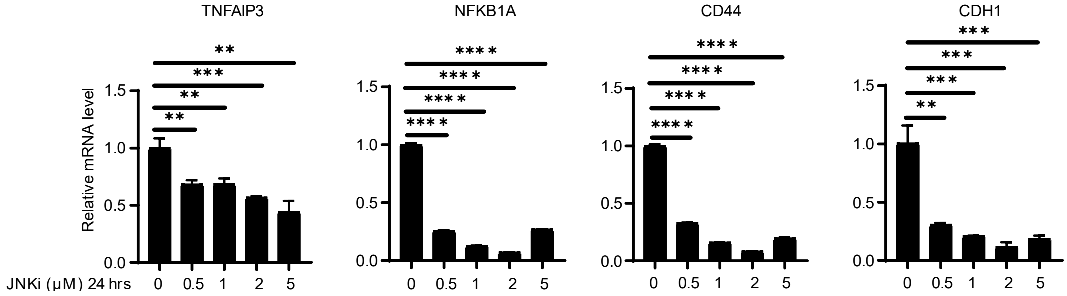

Supplementary Figure 3. Optimization of concentration and time gradient for JNKi treatment of U87 cells. Two-tailed unpaired student's *t*-test was performed, Error bars ± SD, ** $P< 0.01$, *** $P< 0.001$, **** $P< 0.0001$, n=3.

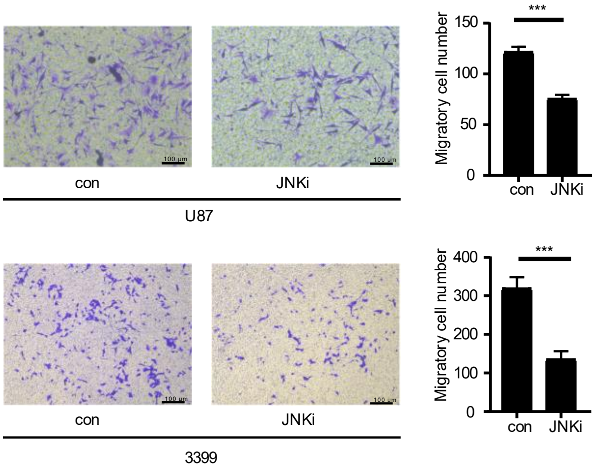

Supplementary Figure 4. Inhibition of JUN activation suppresses the maintenance of MES features. Transwell assays were performed using U87 (a human MES cell line) and 3399 cells (a home-made mouse MES cell line induced with kRas ^{mu}/P53$^{-/-}$ adenovirus) treated with or without JNK-IN-8. Migrated cell was counted and compared. Images are representatives of three independent experiments. Scale bar 100 μm. Two-tailed unpaired student's *t*-test was performed and erro bars mean ± SD, *** $P< 0.001$, n=3.
